# Differential Regulation of Human Bone Marrow Mesenchymal Stromal Cell Chondrogenesis by Hypoxia Inducible Factor‐1α Hydroxylase Inhibitors

**DOI:** 10.1002/stem.2844

**Published:** 2018-06-08

**Authors:** Dheraj K. Taheem, Daniel A. Foyt, Sandra Loaiza, Silvia A. Ferreira, Dusko Ilic, Holger W. Auner, Agamemnon E. Grigoriadis, Gavin Jell, Eileen Gentleman

**Affiliations:** ^1^ Centre for Craniofacial and Regenerative Biology Women's Health Academic Centre KHP, King's College London London United Kingdom; ^2^ Cancer Cell Protein Metabolism Group, Department of Medicine Imperial College London London United Kingdom; ^3^ Division of Women's Health Women's Health Academic Centre KHP, King's College London London United Kingdom; ^4^ Division of Surgery & Interventional Science University College London London United Kingdom

**Keywords:** Bone marrow stromal cells (BMSCs), Cell signaling, Chondrogenesis, Differentiation, Hypoxia, Mesenchymal stem cells (MSCs), Tissue regeneration

## Abstract

The transcriptional profile induced by hypoxia plays important roles in the chondrogenic differentiation of marrow stromal/stem cells (MSC) and is mediated by the hypoxia inducible factor (HIF) complex. However, various compounds can also stabilize HIF's oxygen‐responsive element, HIF‐1α, at normoxia and mimic many hypoxia‐induced cellular responses. Such compounds may prove efficacious in cartilage tissue engineering, where microenvironmental cues may mediate functional tissue formation. Here, we investigated three HIF‐stabilizing compounds, which each have distinct mechanisms of action, to understand how they differentially influenced the chondrogenesis of human bone marrow‐derived MSC (hBM‐MSC) in vitro. hBM‐MSCs were chondrogenically‐induced in transforming growth factor‐β3‐containing media in the presence of HIF‐stabilizing compounds. HIF‐1α stabilization was assessed by HIF‐1α immunofluorescence staining, expression of HIF target and articular chondrocyte specific genes by quantitative polymerase chain reaction, and cartilage‐like extracellular matrix production by immunofluorescence and histochemical staining. We demonstrate that all three compounds induced similar levels of HIF‐1α nuclear localization. However, while the 2‐oxoglutarate analog dimethyloxalylglycine (DMOG) promoted upregulation of a selection of HIF target genes, desferrioxamine (DFX) and cobalt chloride (CoCl_2_), compounds that chelate or compete with divalent iron (Fe^2+^), respectively, did not. Moreover, DMOG induced a more chondrogenic transcriptional profile, which was abolished by Acriflavine, an inhibitor of HIF‐1α‐HIF‐β binding, while the chondrogenic effects of DFX and CoCl_2_ were more limited. Together, these data suggest that HIF‐1α function during hBM‐MSC chondrogenesis may be regulated by mechanisms with a greater dependence on 2‐oxoglutarate than Fe^2+^ availability. These results may have important implications for understanding cartilage disease and developing targeted therapies for cartilage repair. Stem Cells *2018;36:1380–1392*

**Significance Statement**

The repair of damaged cartilage with engineered tissues may be hampered by challenges stemming from the need to provide appropriate environmental cues to progenitor cells. By chemically targeting the hypoxia inducible factor (HIF) complex to mimic the effects of hypoxia, it is shown that the 2‐oxoglutarate (2‐OG) analog dimethyloxalylglycine (DMOG) induces HIF signaling and a more articular chondrocyte‐like expression profile in human bone marrow‐derived mesenchymal stem cells (BM‐MSC) compared with cobalt chloride or desferrioxamine, which reduce divalent iron (Fe^2+^) bioavailability. These observations suggest that human BM‐MSC may rely more on mechanisms that utilize 2‐OG than Fe^2+^ during chondrogenesis and suggest that DMOG could be effective therapeutically for cartilage regeneration.

## Introduction

Acute lesions to the articular cartilage that do not heal may be painful and can progress to osteoarthritis. Conventional treatments such as microfracture or articular chondrocyte implantation are not always effective in mediating repair 1, 2. Tissue engineering strategies that combine cells with bioactive factors and biomaterial scaffolds may allow for de novo articular cartilage formation and provide an alternative therapy for patients 3, 4, 5. However, the provision of cues that can appropriately direct progenitor cell differentiation and tissue formation remain a challenge.

One of the regulatory factors controlling articular cartilage development is the cellular response to physiological hypoxia 6, 7. The cellular response to hypoxia is mediated by the hypoxia inducible factor 8 pathway which induces expression of hypoxia‐responsive genes 9. At normoxia, the hypoxia inducible factor (HIF) complex is unable to recruit the oxygen‐responsive HIF‐1α subunit, which inhibits expression of genes containing a HIF response element within their promoter regions 10. Under hypoxic conditions, HIF‐1α translocates to the nucleus where it complexes with other components of the HIF complex to initiate transcription of HIF target genes 10. HIF‐1α is central in the formation of articular cartilage during development 6, 7. It also plays essential roles in the differentiation of mesenchymal stem/stromal cells (MSC) 11, 12 and chondroprogenitors 13 into cells capable of producing cartilage‐like extracellular matrix (ECM) 14, 15, 16. Moreover, HIF‐1α is vital in maintaining the articular phenotype of differentiated chondrocytes and inhibiting hypertrophic differentiation 17.

Two hydroxylases, prolyl hydroxylase 2 (PHD2) and factor inhibiting HIF (FIH), regulate the participation of HIF‐1α in the HIF complex 18, 19. Each catalyzes the hydroxylation of specific residues on HIF‐1α by utilizing molecular oxygen (O_2_) as a substrate together with ascorbic acid, iron (Fe^2+^) and 2‐oxoglutarate (2‐OG). PHD2‐mediated proline hydroxylation results in ubiquitination of HIF‐1α and its subsequent proteasomal degradation, whereas asparagine hydroxylation by FIH prevents HIF‐1α from binding to the co‐factor, p300 in the HIF complex 18. Under hypoxic conditions, the lack of oxygen reduces PHD2 and FIH activity, enabling HIF‐1α to accumulate in the nucleus and form an active transcriptional complex with co‐factors at the promoter regions of HIF target genes.

The importance of hypoxia and HIF in cartilage development and maintenance point toward its potential utility in cartilage tissue engineering strategies. Indeed, chemical agents that upregulate HIF have been shown to drive the chondrogenic differentiation of MSC and promote articular chondrocytes to produce a cartilage‐like ECM 12, 20, 21. However, studies which compare the efficacy of different HIF‐stimulating compounds in driving the chondrogenesis of human bone marrow‐derived mesenchymal stem cells (hBM‐MSC) compared with standard protocols which utilize transforming growth factor‐β (TGF‐β), are lacking.

**Figure 1:**
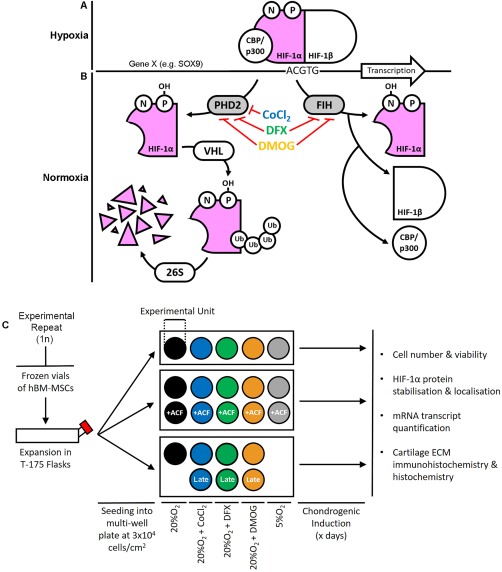
Schematics highlighting the role of hydroxylase inhibitors in regulation of HIF‐1α‐mediated transcription and the study experimental design. **(A):** Under hypoxic conditions, HIF‐1α forms an active transcription complex with HIF‐1β and co‐factors such as CBP/p300. This HIF complex then binds to the promoter regions of target genes at the HIF‐response element sites, inducing transcription. **(B):** At normoxia, two hydroxylases—PHD2 and FIH, utilize oxygen and other substrates to hydroxylate HIF‐1α which promotes its degradation and inhibits binding by CBP/p300. Here, we aimed to stabilize HIF‐1α at normoxia by inhibiting the hydroxylases with CoCl_2_, DFX or DMOG. **(C):** Experiment design. To produce each biological replicate, hBM‐MSCs were thawed and expanded to passage 5 before re‐seeding at a density of 3 × 10^4^ cell per cm^2^ in multi‐well plates. Each well or set of wells was assigned to a specific condition: 20%O_2_, 20%O_2_+CoCl_2_, 20%O_2_+DFX, 20%O_2_+DMOG, or 5%O_2_. Separate experiments included each HIF‐stabilizing compound in the presence or absence of ACF, and a comparison of late with constitutive exposure. In each condition, cultures were chondrogenically differentiated before assays at the time points specified in the legend of each figure. Abbreviations: ACF, Acriflavine; CoCl_2_, cobalt chloride; DFX, desferrioxamine; DMOG, dimethyloxalylglycine; ECM, extracellular matrix; FIH, factor inhibiting hypoxia inducible factor; hBM‐MSCs, human bone marrow‐derived mesenchymal stem cells; HIF, hypoxia inducible factor; PHD2, prolyl hydroxylase 2.

Therefore, we compared the effects of three hydroxylase inhibitors on the chondrogenic differentiation of hBM‐MSC. Dimethyloxalylglycine (DMOG) strongly binds to the 2‐OG binding pocket of both FIH and PHD2, acting as a competitive inhibitor 22; desferrioxamine (DFX) sequesters intracellular Fe^2+^, which is required by FIH and PHD2 23, and thereby reduces their activity; and cobalt chloride (CoCl_2_) competes with Fe^2+^ by directly binding to the PHD2 active site 24. We chose these agents because they cover the main classes of HIF‐stimulating compounds, and as such, upregulate HIF‐1α via distinct mechanisms (Fig. 1A, 1B). These compounds are also the most widely studied for chemically regulating HIF and may shed light on key regulatory elements of hypoxic signaling during chondrogenesis. Moreover, investigating the PHD2/FIH inhibitors during hBM‐MSC chondrogenesis may aid our understanding of the pathophysiology of degenerative diseases such as osteoarthritis 8, for which HIF‐1α is known to play a protective role 25.

Here, we show that while CoCl_2_, DFX, and DMOG all induce similar levels of HIF‐1α stabilization, only DMOG strongly enhances HIF‐mediated transcription of key chondrogenic genes. Nevertheless, DMOG negatively impacted the production of Collagen Type II and glycosaminoglycans (GAGs), which could be alleviated by only exposing cells to the compound during the latter stages of chondrogenesis. Together, these observations highlight the potential importance of mechanisms which utilize 2‐OG compared with Fe^2+^ for the transcriptional control of HIF target genes during hBM‐MSC chondrogenesis. They also suggest that 2‐OG inhibitors may better promote a chondrogenic transcriptome compared with either DFX or CoCl_2_. These observations may inform on improved, targeted strategies for stimulating cartilage ECM formation in tissue engineering‐based therapies.

## Materials and Methods

### Isolation and Expansion of hBM‐MSC

hBM‐MSCs were isolated from bone marrow aspirates collected from the iliac crest of healthy pediatric donors, with informed consent from their parents or guardians. Cells were seeded in CellSTACK (Corning, Sigma Aldrich, UK) culture chambers at 10–25 × 10^6^/636 cm^2^ and cultured in αMEM supplemented with human platelet lysate (Stemulate, Cook Medical, USA). At 90%–100% confluency, cells were passaged and seeded at 5,000 cells per cm^2^. For immunophenotyping of hBM‐MSCs, the following antibodies were used in conjunction with a FACSCalibur analyzer (BD Biosciences, UK): CD90‐FITC, CD105‐APC, CD73‐PE, CD34‐PE, and CD45‐FITC (all from BD Biosciences). All human tissue was approved for use by the UK National Research Ethics Service (12/WA/0196) and was collected by the National Institute for Health Research, which is supported by the Imperial College Healthcare Tissue Bank (HTA license 12275). Cultures were found to express CD90, CD105, CD73 and not express hematopoietic markers CD34 and CD45 26 (data not shown). hBM‐MSCs were expanded in growth media (GM; αMEM + 10% fetal bovine serum [FBS], Thermo Fisher Scientific, UK) under standard conditions (5% CO_2_).

### Chondrogenic Induction of hBM‐MSC

hBM‐MSCs were expanded to passage 5 in GM under standard culture conditions before cryopreservation in a solution composed of 10% dimethyl sulfoxide, Sigma‐Aldrich, 40% FBS, and 50% GM. Cells were stored in liquid nitrogen prior to use. For chondrogenic induction experiments, cryovials of hBM‐MSCs were thawed in GM and grown to confluence before plating at 3 × 10^4^/cm^2^ into multi‐well tissue culture plates. Cultures were incubated for 24 hours in GM prior to induction using standard chondrogenic differentiation media (CDM). See Figure 1C for experimental plan. Cells were differentiated as monolayers to prevent the formation of a local hypoxic microenvironment independent of experimental conditions (physiological or chemically induced hypoxia). Indeed, while pellet/micromass cultures may be more conducive for chondrogenesis, the bioavailability of oxygen may vary between cells at the periphery and center of such cultures. CDM consisted of High Glucose Dulbecco's modified Eagle medium (Sigma‐Aldrich) + 2 mM l‐Glutamine (Thermo Fisher Scientific) + 100 nM Dexamethasome (Sigma‐Aldrich) + 1% Insulin, Transferrin, Selenium Solution (Thermo Fisher Scientific) + 1% Antibiotic Antimycotic solution (Sigma‐Aldrich) + 50 μg/ml Ascorbic acid‐2‐phosphate (Sigma‐Aldrich) + 40 μg/ml l‐proline (Sigma Aldrich) + 10 ng/ml TGF‐β_3_ (Peprotech). CDM was supplemented with HIF‐stabilizing compounds (Sigma‐Aldrich): 100 μM CoCl_2_, 50 μM DFX, and 200 μM DMOG, or incubated in un‐supplemented CDM at hypoxia (5%O_2_) or normoxia. To achieve HIF‐1α inhibition, media was further supplemented with 500 nM Acriflavine (ACF; Santa Cruz Biotechnology, USA).

### Neutral Red Viability Assay

Neutral red dye (Sigma‐Aldrich) dissolved in cell culture medium was incubated with differentiating hBM‐MSC for 2 hours before fixation in 0.1% Calcium Chloride + 0.5% paraformaldehyde (both from Sigma‐Aldrich). Dye retained by hBM‐MSC was solubilized in 1% acetic acid + 50% ethanol (both from Sigma‐Aldrich). Quantification of solubilized Neutral Red was then performed on an absorbance spectrophotometer at 540 nm.

### PicoGreen Assay

Samples were snap‐frozen at −80°C and digested in 400 μg/ml Papain Buffer at 65°C for 18 hours. Double stranded deoxyribonucleic acid (dsDNA) content in papain‐digested cultures was quantified using a PicoGreen kit (Thermo Fisher Scientific). A linear relationship was observed between hBM‐MSC number and dsDNA content.

### Sodium Dodecyl Sulfate‐PAGE and Western Blotting

Following 24‐hours of culture, cells were lysed in sodium dodecyl sulfate (SDS) buffer and protein was quantified using a Bicinchoninic Acid assay (Thermo Fisher Scientific). Lysates were run on polyacrylmide gels (Biorad, UK) and transferred using the Trans‐Blot Turbo Transfer System (Biorad). HIF**‐**1α and housekeeping protein β‐Actin were bound by primary antibodies (H‐206; Santa Cruz Biotechnology and ab8227; Abcam, UK). Signal detection produced between a horseradish peroxidase‐conjugated secondary antibody (sc‐2004; Santa Cruz) and the Chemiluminescent ECL substrate (Biorad) were detected on a Chemidoc Touch imaging platform (Biorad). HIF**‐**1α and protein levels were generated by densitometric analysis with ImageJ and normalized to that of β‐Actin.

### Quantitative Polymerase Chain Reaction

RNA was extracted using the RNeasy Mini Kit (Qiagen, DE). Hundred nanograms of RNA per sample was reverse transcribed using M‐MLV Reverse Transcriptase (Promega, UK) and cDNA was amplified using quantitative polymerase chain reactions (qPCR) in a CFX384 (Biorad). Brilliant III Ultra‐Fast SYBR Green QPCR Master Mix (Agilent, USA) was used in conjunction with primers specific to genes of interest. Primer sequences are shown in Supporting Information Table S1. All primers produced a linear relationship between template concentration and Ct value. Reaction efficiencies were confirmed to lie between 90 and 110%. Raw Ct values were converted to transcript copy number by the relative standard curve method of analysis, and expression levels were normalized to that of *RPL13A*. Following normalization to the housekeeping gene, expression levels were then normalized to that of the untreated control to determine fold change in expression induced by each treatment.

### Immunofluorescence Staining

Cultures were fixed in 4% (wt/vol) paraformaldehyde for 15 minutes. HIF‐1α and Collagen Type II were then detected using H‐206 (Santa Cruz) and ab34712 (Abcam), respectively, overnight, following blocking with (10%) goat serum (Sigma‐Aldrich) for 60 minutes and permeabilization in 0.1% (vol/vol) Triton X‐100 solution (Sigma Aldrich) for 60 minutes, both at room temperature (RT). Collagen Type X was detected using ab49945 (Abcam) at a 1:250 dilution overnight. Rabbit‐derived primary antibodies were visualized with ab150077 (Abcam) after staining for 60 minutes at RT at dilutions of 1:100 and 1:200 for Collagen Type II and HIF‐1α, respectively. Mouse‐derived primary antibodies were detected with biotin (ab6788, Abcam) and Streptavidin (S11223, Thermo Fisher Scientific) both at 1:350 for 60 minutes. Cultures were counterstained with 0.1 μg/ml DAPI for 60 minutes to visualize nuclei and fluorescence was imaged on an Axiovert200M microscope (Zeiss, DE). The images in Supporting Information Figure S1 confirm that signal was due to each primary antibody and not background fluorescence or nonspecific binding of the secondary antibody.

### Alcian Blue Staining

Cultures fixed in 4% paraformaldehyde were stained with 1% Alcian blue solution, pH 1.0 (Sigma‐Aldrich) prepared in 0.1N HCl. Hematoxylin (Vector Laboratories, UK) was used to visualize cell nuclei and staining was imaged on an Axiovert200M microscope (Zeiss).

### Glycosaminoglycan Quantification

At day 21 of chondrogenesis, cultures were washed in phosphate buffered saline and frozen at −80°C before their digestion in 400 μg/ml Papain buffer (Sigma‐Aldrich) supplemented with 0.2M Sodium Phosphate + 5 mM Ethylenediaminetetraacetic acid + 5 mM l‐Cysteine (all Sigma‐Aldrich) at 65°C for 18 hours. GAGs were quantified from Papain‐digested lysates using a Blyscan GAG assay kit (Biocolor, UK) in which GAGs were dyed with 1,9‐dimethyl‐methylene blue and subsequently dissociated with Propan‐1‐ol solution before quantification on an absorbance spectrophotometer at 640 nm. Values were normalized to levels of dsDNA, which were quantified using the PicoGreen assay.

### Immunofluorescence Quantification

Immunofluorescence images were captured using identical gain, exposure, and offset for all conditions in each experiment. These were determined with positive controls that expressed the antigen of interest, and negative controls in which the primary antibody was omitted (Supporting Information Fig. S1). The same threshold fluorescence intensity for images of all conditions within an experiment was set, below which the signal produced was negated as background. The signal produced above the threshold was regarded as bona fide protein detection and was used to create a binary representation of each image. The percentage of immunofluorescence staining present within a specified area was then determined.

### Statistical Analysis

All statistical analyses were performed in Prism7 (GraphPad, USA) with the Mann‐Whitney test used to compare two conditions and Kruskal‐Wallis with Dunn's Correction for multiple condition comparisons. Nonparametric tests were used as we were unable to demonstrate normality in all datasets. *marks all differences that were statistically significant (*p* < .05).

## Results

### Hypoxia Promotes HIF Stabilization and a More Articular Cartilage‐Like Cell Phenotype

**Figure 2:**
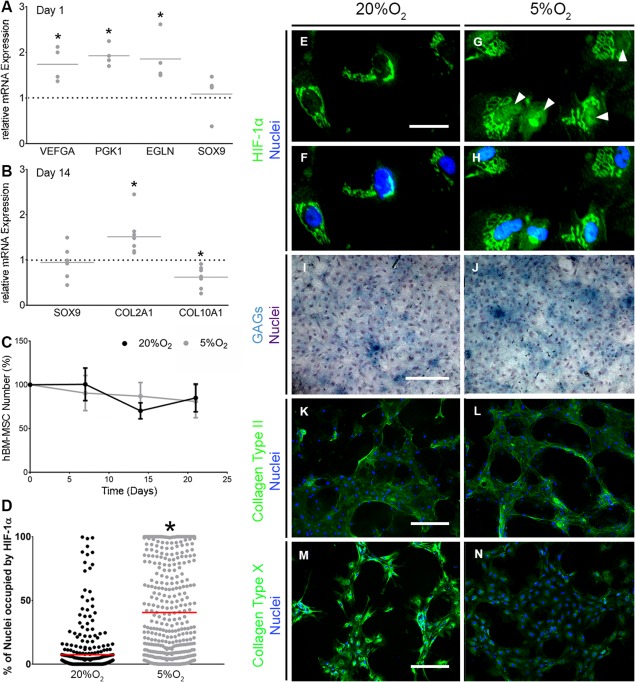
Hypoxia induces HIF‐1α nuclear localization and promotes an articular chondrocyte‐like phenotype. **(A, B):** gene expression of *VEGFA*, *EGLN* and *PGK1*, and *SOX9* (*n* = 4) at day 1 (A) and *SOX9*, *COL2A1,* and *COL10A1* (*n* = 8) at day 14 of chondrogenesis (B). Values plotted are fold change in response to 5%O_2_ compared with 20%O_2_, which is represented by the horizontal dotted line. The solid gray line represents the mean. *denotes *p* < .05 compared with 20%O_2_. **(C):** Human bone marrow‐derived mesenchymal stem cells number throughout chondrogenesis as determined by PicoGreen assay (*n* = 3). Values are normalized to cell number at day 0 and error bars show standard error of the mean. **(D):** Quantification of nuclear HIF‐1α immunofluorescence in **(F–H)** at day 1 of chondrogenesis (*n* = 3). Each value represents the percentage of a single nucleus that is occupied by HIF‐1α. The red horizontal line represents the mean with *p < .05 compared with 20%O_2_. (**E–H)** HIF‐1α immunofluorescent staining. Scale bar = 50 μm. Representative images of 3 independent repeats. Images were cropped and magnified to visualize localization of HIF‐1α. **(I, J):** Alcian blue staining for GAGs with hematoxylin counterstain at day 21 of chondrogenesis (*n* = 3). Scale bar = 400 μm. **(K–N):** Collagen Type II (K, L) and X (M, N) immunofluorescence staining at day 21 of chondrogenesis (*n* = 3). Scale bar = 400 μm. Brightness and contrast were adjusted to an equal degree between all conditions. Abbreviations: GAGs, glycosaminoglycans; HIF, hypoxia inducible factor.

It is well established that hBM‐MSCs can be chondrogenically differentiated with transforming growth factor β_3_ (TGF‐β_3_) ligands. Therefore, we first aimed to determine if chondrogenesis could be further enhanced by culture under hypoxic conditions, as previously reported 11. Hypoxia increased expression of a selection of known HIF target genes including *VEGFA*, *EGLN*, and *PGK1* (all *p =* .0286) 27, 28, 29 compared with that in hBM‐MSC cultured under normoxic conditions (Fig. 2A). These observations were in line with previous studies which have similarly shown a rapid (24 hours) upregulation of HIF and HIF‐mediated transcription in response to hypoxia under chondrogenic conditions 11. However, 5%O_2_ did not significantly affect expression of *SOX9* (Fig. 2A, 2B; *p* = .1), the master transcriptional regulator of chondrogenesis 30, after either 1 or 14 days in culture.

At day 14, we observed upregulation of expression of the gene for the articular cartilage ECM component Collagen Type II (*COL2A1, p =* .0002), and downregulation of the hypertrophic marker Collagen Type X 31 (*COL10A1, p =* .0006) under hypoxic conditions compared with that at normoxia (Fig. 2B). *COL2A1* and *COL10A1* are targets of transcription factors SOX9 and RUNX2, respectively, and are known to be regulated as the chondrogenic differentiation of MSC proceeds 11. Culture for 21 days under hypoxic conditions did not affect cell viability or proliferation (Fig. 2C). However, as expected, we did observe increased HIF‐1α nuclear localization (*p <* .0001) in hypoxic compared with normoxic cultures (Fig. 2D–2H). Hypoxia also increased Alcian Blue staining of GAGs (Fig. 2I, 2J), but did not affect the immuno‐detection of Collagen Type II protein (Fig. 2K, 2L). Nevertheless, we did detect a decrease in staining for Collagen Type X (Fig. 2M, 2N), consistent with hypoxia's inhibitory role to chondrocyte hypertrophy 17. Together, these observations confirmed that culture under hypoxic conditions in the presence of TGF‐β_3_ promoted an articular chondrocyte‐like phenotype that was conducive for articular cartilage ECM rather than hypertrophic cartilage formation. This effect appeared to not require a corresponding upregulation of *SOX9*, but instead correlated with increased immunostaining for HIF‐1α, upregulation of select HIF target genes *VEGFA*, *EGLN*, and *PGK1*, and increased HIF‐1α nuclear localization.

### CoCl_2_, DFX, and DMOG Induce HIF‐1α Localization, but Only DMOG Strongly Upregulates HIF Targets *VEGFA*, *PGK1,* and *ELGN*

**Figure 3:**
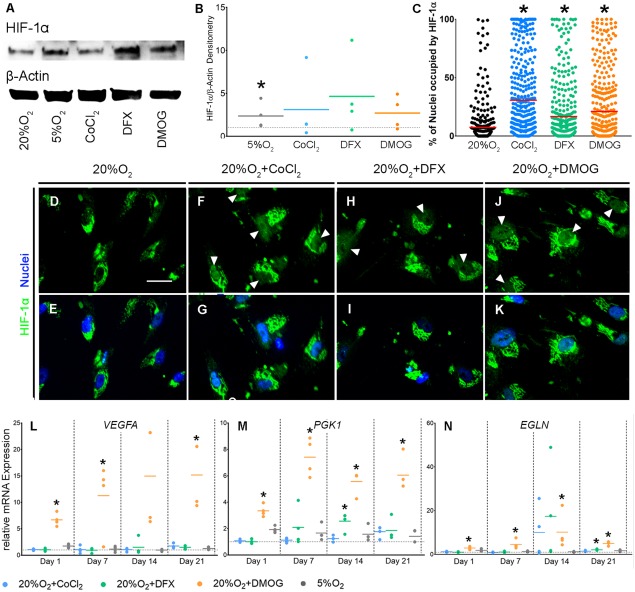
CoCl_2_, DFX, and DMOG increase nuclear localization of HIF‐1α but only DMOG induces stable upregulation of HIF targets. **(A, B):** Detection of HIF‐1α and housekeeping protein, β‐Actin by Western Blot analysis of whole‐cell lysates from hBM‐MSCs at day 1 of chondrogenesis (A; *n* = 4). Western blots were quantified and normalized to levels of β‐Actin (B; *n* = 4). Values are magnitude difference compared with the no treatment control, which is represented by the horizontal dotted line. Solid colored lines are the mean for each condition. **(C):** Quantification of nuclear HIF‐1α immunofluorescence at day 1 of chondrogenesis (*n* = 4). Each value represents the percentage of a single nucleus that is occupied by HIF‐1α. The red horizontal line represents the mean and *p < .05 compared with 20%O_2_. **(D–K):** HIF‐1α immunofluorescence staining at day 1 of chondrogenesis (*n* = 4). Scale bar = 50 μm. Images were cropped and magnified to visualize localization of HIF‐1α. Brightness and contrast were adjusted to an equal degree between all conditions. **(L–N):** Gene expression of *VEGFA*, *PGK1,* and *EGLN* due to CoCl_2_, DFX, DMOG, and 5%O_2_ (*n* = 4). Values are fold change compared with the no treatment control represented by the horizontal dotted line. Solid colored lines represent the mean for each condition. *p < .05 compared with 20%O_2_ at the same time point. Abbreviations: CoCl_2_, cobalt chloride; DFX, desferrioxamine, DMOG, dimethyloxalylglycine; HIF, hypoxia inducible factor.

Having determined that hypoxia promoted HIF‐1α stabilization and expression of *VEGFA*, *EGLN*, and *PGK1*, we next aimed to determine if inhibitors of the hydroxylases PHD2 and FIH would have a similar effect on hBM‐MSC cultured under normoxic conditions. We first determined appropriate doses for the hydroxylase inhibitors DMOG, DFX, and CoCl_2_ by confirming that concentrations of each used extensively in the literature 22, 24, 32, 33, 34 were nontoxic to hBM‐MSC over 21 days of chondrogenic differentiation (Supporting Information Figs. S2, S3). Next, we confirmed that each could stabilize HIF by carrying out Western blots for HIF‐1α in whole‐cell lysates after 24 hours, as HIF is known to be rapidly induced in response to PHD2/FIH inhibition 22. Levels of HIF‐1α protein were significantly increased in cells cultured under hypoxic conditions (*p =* .0286); however, despite trends for increased levels of HIF‐1α after treatment with HIF stabilizing compounds, we failed to detect statistically significant differences (*p =* .314) compared with controls (Fig. 3A, 3B). Nonetheless, nuclear localization of HIF‐1α was enhanced compared with controls (*p* ≤ .0001) in response to treatment with all three compounds (Fig. 3C–3K).

We then examined the effects of the hydroxylase inhibitors on HIF target gene expression. DMOG significantly and consistently upregulated expression of *VEGFA* (*p =* day 1: .0073, day 7: .0470, day 21: .0005), *PGK1 (p =* day 1: .0073, day 7: .0013, day 14: .0013, day 21: .0031), and *EGLN* (*p =* day 1: .0108, day 7: .0332, day 14: .0470, day 21: .0005) (Fig. 3L–3N). However, the effects of CoCl_2_ and DFX were more subtle, and we only observed upregulation of *PGK1* expression at day 14 (*p* = .0391) and *EGLN* at day 21 (*p =* .0396) in response to DFX. These observations show that while CoCl_2_, DFX, and DMOG all affect HIF‐1α stabilization, only DMOG strongly upregulated expression of a selection of HIF target genes. This suggests that DMOG more potently enhanced HIF activity compared with DFX or CoCl_2_.

### DMOG Stimulates hBM‐MSC to Adopt an Articular Chondrocyte‐Like Transcriptional Profile

**Figure 4:**
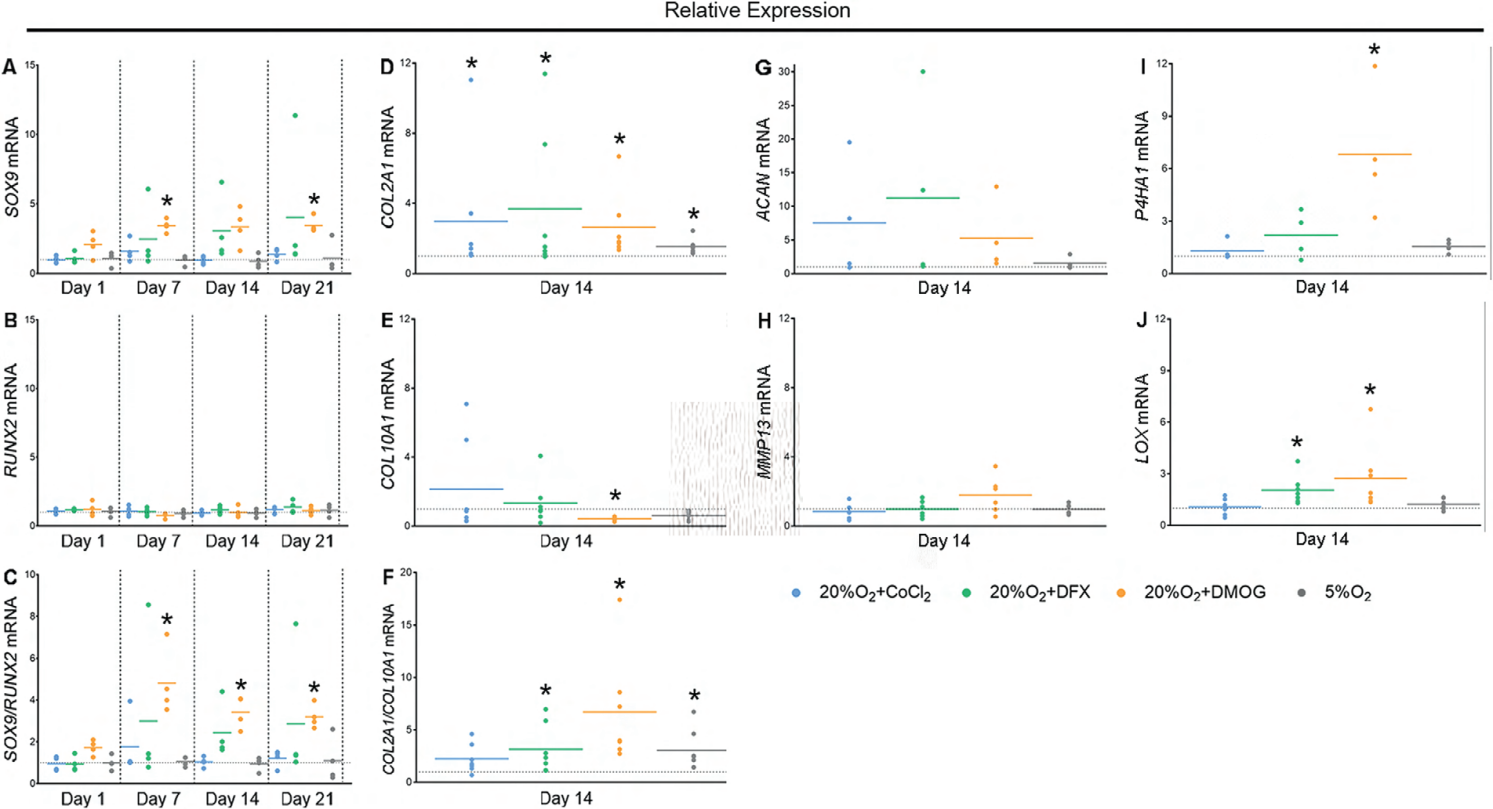
DMOG induces a chondrogenic transcriptional profile and inhibits markers of osteoblastic and hypertrophic differentiation but also reduces the formation of a cartilage‐like ECM. **(A–J):** Gene expression of *SOX9* (A; *n* = 4), *RUNX2* (B; *n* = 4) and *SOX9*/RUNX2 (C; *n* = 4), *COL2A1* (D; *n* = 7), *COL10A1* (E; *n* = 7), *COL2A1*/*COL10A1* (F; *n* = 7), *ACAN* (G; *n* = 4), *MMP13* (H; *n* = 7), *P4HA1* (I; *n* = 4), and *LOX* (J; *n* = 7) throughout chondrogenesis due to treatment with CoCl_2_, DFX, DMOG, and 5%O_2_. Values are fold change compared with the no‐treatment control represented by the horizontal dotted line. Solid colored lines are the mean for each condition. *p < .05 compared with 20%O_2_ at the same time point. Abbreviations: CoCl_2_, cobalt chloride; DFX, desferrioxamine, DMOG, dimethyloxalylglycine.

As all HIF mimetics stabilized HIF‐1α and DMOG also upregulated expression of HIF target genes, we next investigated the effect of these compounds on chondrogenic gene expression. DMOG treatment upregulated *SOX9* gene expression after 7 (*p* = .0159) and 21 (*p* = .0332) days in culture (Fig. 4A), while *RUNX2*, a key regulator of osteogenesis 35, was unaffected under all conditions (Fig. 4B). This resulted in a DMOG‐mediated increase in the *SOX9* to *RUNX2* expression ratio throughout differentiation (Fig. 4C; *p =* day 7: .0192, day 14: .0398, day 21: .0159). All inhibitors upregulated expression of *COL2A1* (Fig. 4D; *p* = CoCl_2_: .0280, DFX: .0180, DMOG: .0008) with DMOG significantly downregulating *COL10A1* (Fig. 4E; *p* = .0037) leading to an increased *COL2A1*/*COL10A1* mRNA ratio due to DFX and DMOG (Fig. 4F; *p* = DFX: .0259, DMOG: <.0001). *ACAN*, which encodes the gene for Aggrecan, the most abundant proteoglycan in cartilage 36, was not upregulated by any treatment (Fig. 4G); and *MMP13*, whose product contributes to an osteoarthritic chondrocyte phenotype 37, was similarly unaffected (Fig. 4H). The articular cartilage phenotype is marked by appropriate post‐translational modifications of secreted collagen by enzymes encoded by *P4HA1* and *LOX* 14, 15. DMOG strongly upregulated both *P4HA1* (Fig. 4I; *p* = .0039) and *LOX* (Fig. 4J; *p* = .0027) expression, while DFX only upregulated *LOX* (*p* = .0056) and CoCl_2_ had no significant effects. Taken together, these observations demonstrate that DMOG upregulated transcriptional regulators of chondrogenesis and genes involved in cartilage ECM formation, while the effects of CoCl_2_ and DFX were more limited.

### DMOG Inhibits Incorporation of Collagen Type II, Type X, and GAGs into the Cell‐Secreted ECM

**Figure 5:**
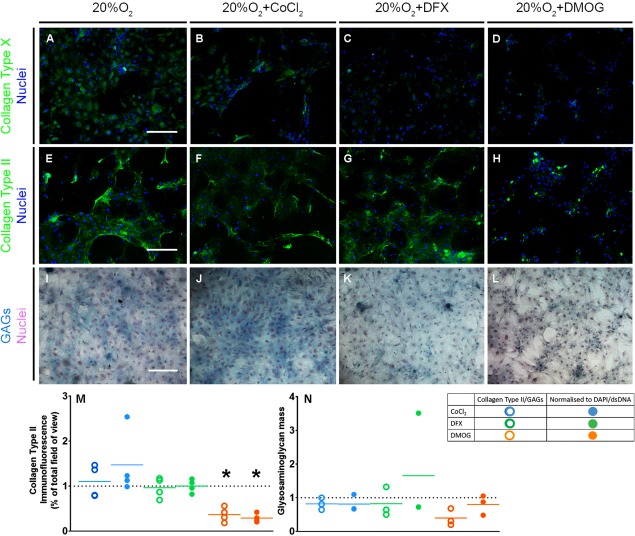
DMOG reduces the formation of a cartilage‐like ECM. **(A–H):** Collagen Type X (A–D) and II (E–H) immunofluorescence staining at day 21 of chondrogenesis (*n* = 3). Scale bar = 400 μm. Brightness and contrast were adjusted to an equal degree between all conditions. **(I–L):** Alcian blue staining for GAGs with hematoxylin counterstain at day 21 of chondrogenesis (*n* = 3). Scale bar = 400 μm. **(M, N):** Quantification of Collagen Type II immunofluorescence (M) and glycosaminoglycans (N) at day 21 of chondrogenesis (*n* = 4) without and with normalization to DAPI‐immunofluorescence/double‐stranded DNA. Values are fold change compared with the no‐treatment control represented by the horizontal dotted line. Solid colored lines represent means for each condition. *p < .05 compared with 20%O_2_. Abbreviations: CoCl_2_, cobalt chloride; DFX, desferrioxamine, DMOG, dimethyloxalylglycine; GAGs, glycosaminoglycans.

As treatment with DMOG regulated the expression of genes associated with a chondrocyte phenotype, we next asked if this influenced cartilage‐like matrix formation. In line with changes in gene expression, hBM‐MSC treated with DMOG for 21 days showed little to no staining for Collagen Type X compared with controls (Fig. 5A, 5D). We observed a similar effect in both DFX‐ and CoCl_2_‐treated cultures (Fig. 5B, 5C). However, while CoCl_2_‐ and DFX‐treated cultures showed similar levels of staining for Collagen Type II as controls (Fig. 5E–5G), DMOG‐treated cultures showed only sparse staining (Fig. 5H). This was confirmed by quantification of Collagen Type II immunofluorescence both without (*p* = .0286) and with normalization to cell number which indicated reduced Collagen Type II production per cell (Fig. 5M; *p* = .0286). Alcian blue staining confirmed these observations as DMOG‐treated cultures showed fewer GAG‐positive areas than the other groups, although quantitative differences in staining on a per cell basis were not significant (Fig. 5I–5L, 5N). Overall, DMOG appeared to reduce the total amount of cartilage‐like ECM that cells formed in their immediate extracellular space.

### HIF‐1α Mediates DMOG's Induction of an Articular Chondrocyte Transcriptional Profile

**Figure 6:**
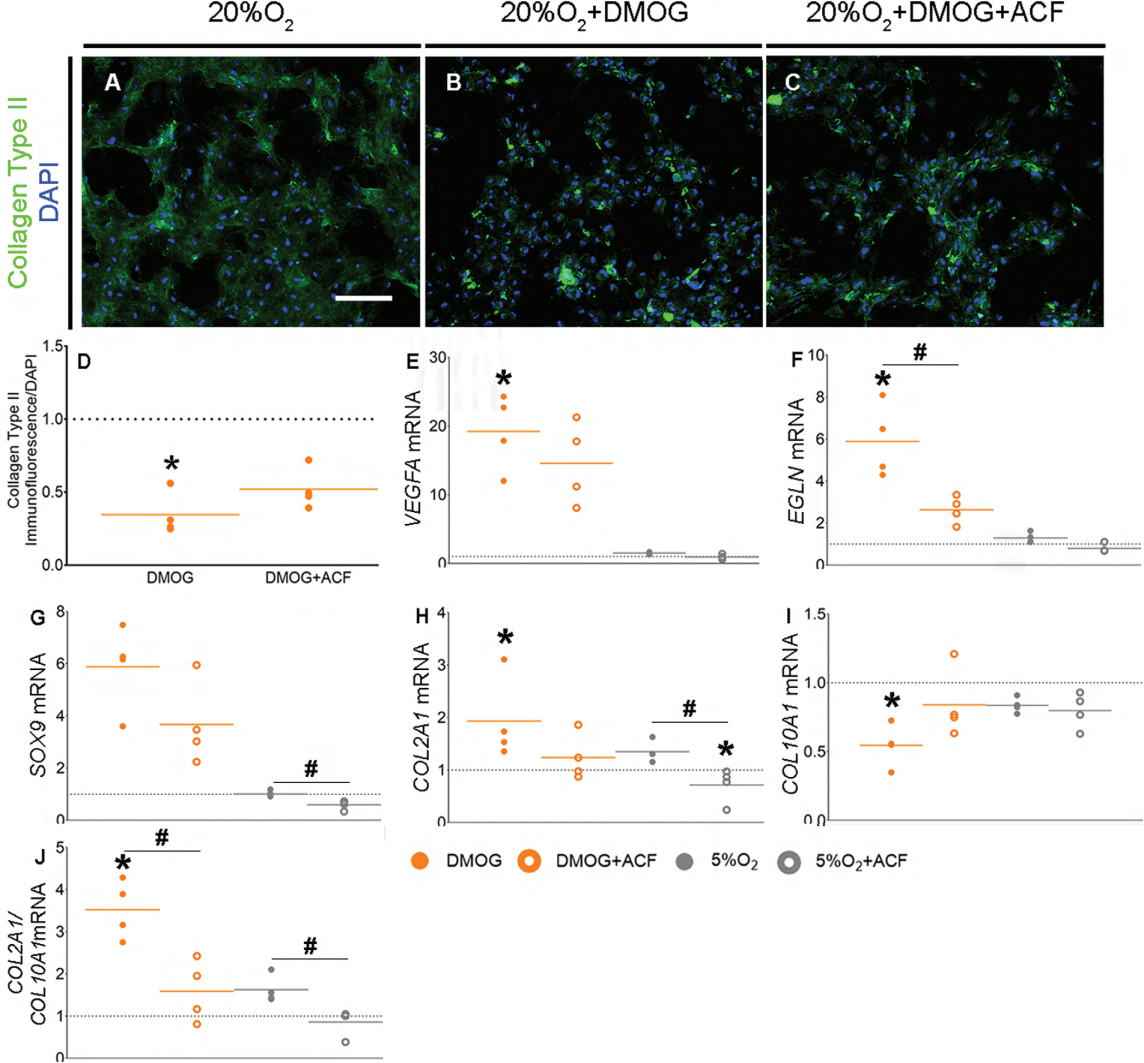
Inhibition of HIF‐1α reduces the DMOG‐mediated upregulation of a chondrogenic transcriptional profile. **(A–C)**: Collagen Type II immunofluorescence staining at day 14 of chondrogenesis. Scale bar = 400 μm (*n* = 4). Brightness and contrast were adjusted to an equal degree between all conditions. **(D)**: Quantification of Collagen Type II immunofluorescence at day 14 of chondrogenic induction, normalized to equivalent DAPI‐immunofluorescence (*n* = 4). Values are fold change compared with the no‐treatment control represented by the horizontal dotted line. Orange lines represent means for each condition. *p < .05 compared with 20%O_2_. **(E–J)**: Gene expression of *VEGFA* (E) and *EGLN* (F), *SOX9* (G), *COL2A1* (H), *COL10A1* (I), and *COL2A1*/*COL10A1* (J) at day 14 of chondrogenesis (*n* = 4) after treatment with DMOG, 5%O_2_, DMOG + Acriflavine, or 5%O_2_ + Acriflavine. Values are fold change compared with the no‐treatment condition represented by the horizontal dotted line. Solid colored lines represent means for each condition. *p < .05 when compared with 20%O_2_, and ^#^ *p* < .05 between +/–ACF conditions within 20%O_2_ + DMOG or 5%O_2_ groups. Abbreviations: ACF, Acriflavine; DMOG, dimethyloxalylglycine.

As DMOG mediated antithetical effects in terms of chondrogenic transcriptional profile and ECM formation, we next aimed to study its mechanism of action. To accomplish this, we supplemented CoCl_2_/DFX/DMOG‐containing CDM with Acriflavine (ACF), an inhibitor of HIF‐1α and HIF‐1β binding 38. ACF abolished the DMOG‐induced upregulation of established HIF targets, but did not affect total cell number during chondrogenesis (Supporting Information Fig. S4A, S4B). Staining for Collagen Type II in DMOG‐treated cultures supplemented with ACF remained sparse (Fig. 6A–6C), but quantitative image analyses showed staining on a per cell basis was no different from controls, while cultures treated with DMOG alone were significantly lower (Fig. 6D; *p* = .0076). This suggests that the inhibitory role of DMOG on Collagen Type II matrix formation may be partly mediated through HIF‐1α activity.

We next asked if DMOG's stimulation of the chondrogenic transcription profile in hBM‐MSC was also mediated through HIF‐1α. ACF abrogated DMOG‐mediated changes in expression of HIF targets, *VEGFA* (Fig. 6E*; p* = ‐ACF: .0132, +ACF: .0772) and *EGLN* (Fig. 6F; *p* = ‐ACF: .0073, +ACF: 0.1232), *COL2A1* (Fig. 6H; *p* = ‐ACF: .0286, +ACF: >.999), *COL10A1* (Fig. 6I; *p* = ‐ACF: .002, +ACF: >.3277), the *COL2A1/COL10A1* ratio (Fig. 6J; *p* = ‐ACF: .0031, +ACF: >05998), and showed a similar trend for *SOX9* expression (Fig. 6G; *p* = ‐ACF: .0772, +ACF: .5348) Interestingly, despite a lack of upregulation of *SOX9* under hypoxic conditions, we observed a negative effect of hypoxia on *SOX9* expression in the presence of ACF (Fig. 6G; *p* = .0286). This is consistent with the observation that ACF reduced the ratio of *COL2A1*/*COL10A1* under hypoxic conditions (Fig. 6J; *p* = .0286) and suggests that hypoxia, via HIF‐1α, does indeed regulate basal levels of chondrogenic targets genes, such as *SOX9*. Overall, these data suggest that HIF‐1α mediated DMOG's effect on the transcriptional profile of chondrogenically induced hBM‐MSC. Moreover, ACF appeared to have a larger effect on DMOG‐mediated transcription than that induced by either CoCl_2_ or DFX (Supporting Information Fig. S4C–S4J).

### Late DMOG Treatment Enhances Chondrogenesis

**Figure 7:**
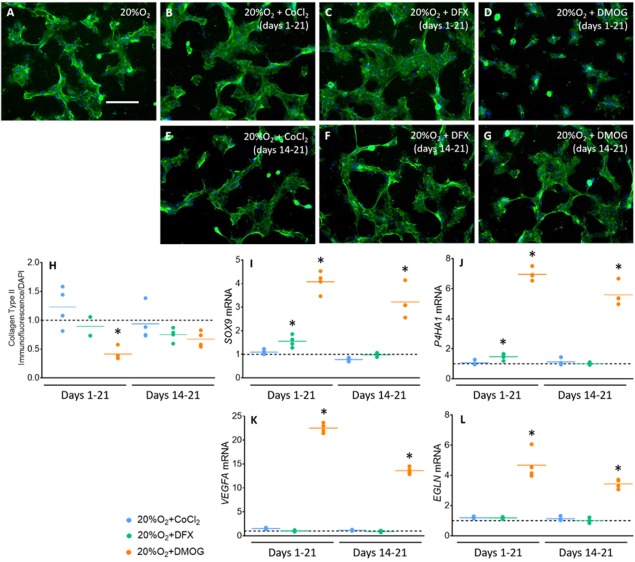
Late DMOG treatment does not inhibit the formation of a Collagen Type II‐rich ECM but does induce a chondrogenic expression profile. **(A–G):** Collagen Type II immunofluorescence staining at day 21 of chondrogenesis after continuous (days 1–21) and late (days 14–21) CoCl_2_, DFX and DMOG treatment (*n* = 4). Scale bar = 400 μm. Representative images from 4 independent repeats shown. Brightness and contrast were adjusted for all channels to an equal degree between all conditions. **(H):** Quantification of Collagen Type II immunofluorescence at day 21 of chondrogenic induction, normalized to equivalent DAPI‐immunofluorescence (*n* = 4). Values are fold change compared with the no‐treatment control represented by the horizontal dotted line. The horizontal, colored lines represent means for each condition. *, *p* < .05 compared with 20%O_2_. **(I–L):** Gene expression of *SOX9* (I), *P4HA1* (J), *VEGFA* (K), and *EGLN* (L) after continuous (days 1–21) and late (days 14–21) CoCl_2_, DFX and DMOG treatment (*n* = 4). Values are normalized to the housekeeping gene *RPL13A* and are fold change compared with the no‐treatment control, represented by the horizontal dotted line. The horizontal colored lines represent the means for each condition. *, *p* < .05 when compared with 20%O_2_. Abbreviations: CoCl_2_, cobalt chloride; DFX, desferrioxamine, DMOG, dimethyloxalylglycine.

As DMOG upregulated chondrogenic transcripts but continuous treatment led to reduced staining for cartilage‐like matrix, we next asked if altering either the length/timing of treatment would influence ECM formation. Therefore, we next treated hBM‐MSC with DMOG, DFX, and CoCl_2_ either continuously (as before) or during late (days 14–21) time periods and analyzed mRNA and protein expression of ECM markers after 21 days. Late DMOG treatment did not negatively affect the secretion of Collagen Type II compared with controls (*p* = .282), as we observed with continuous DMOG treatment (*p* = .0188). This contrasted with treatment with either DFX or CoCl_2_, where both continuous and late treatment had no effect on Collagen Type II secretion (*p* ≥ .9999 for both, Fig. 7A–7H). At the gene expression level, like continuous treatment (*p* = .0023), late exposure to DMOG induced significant upregulation of *SOX9* (Fig. 7I; *p* = .0168). Late DMOG also upregulated expression of *P4HA1* (Fig. 7J; *p* = .0286), and HIF targets *VEGFA* (*p* = .0358, Fig. 7K) and *EGLN* (*p* = .0208, Fig. 7L) as with continuous DMOG treatment (*p = P4HA1*: .0313, *VEGFA*: .0118, *EGLN*: .0088). In contrast, neither continuous nor late CoCl_2_ and DFX treatment significantly affected the expression of these genes, with the exception of continuous DFX treatment on *SOX9* (*p* = .0286; Fig. 7I) and *P4HA1* (*p* = .0286; Fig. 7J). Taken together, late treatment with DMOG induced a similar expression profile to continuous treatment, but without negatively impacting the formation of cartilage‐like ECM.

## Discussion

Hypoxic conditions are known to favor articular cartilage development. The pro‐chondrogenic effects of hypoxia are thought to be mediated primarily through HIF‐1α via the formation of a transcriptionally‐active complex at target genes 7, 12. Therefore, we and others hypothesized that compounds that increase HIF‐1α availability would promote HIF‐mediated chondrogenesis. Previous studies have examined the effect of CoCl_2_ 12, DFX 39, and DMOG 21, 40 in this context. While such studies have cemented the role of HIF‐1α in chondrogenesis, to our knowledge no study has yet examined their comparative effects during cartilage formation or the chondrogenic differentiation of precursors. As the inhibitors have differential mechanisms of action, comparatively studying their effects may have important implications for HIF biology and cartilage regenerative medicine. Indeed, instead of utilizing physiological hypoxia for regenerative medicine, stabilizing the HIF complex under normoxic conditions would remove the complex logistics required for spatial organization of oxygen. This may be particularly valuable in engineering constructs for the repair of full osteochondral defects due to the contrasting oxygen requirements of avascular cartilage and vascularized bone 6. HIF mimetics could also potentially avoid the undesirable HIF‐independent effects of hypoxia such as the unfolded protein response and associated cell stress 41, and could preclude the development of a tolerance to the reduced oxygen levels 42, 43.

In our control conditions, we defined hypoxia as 5%O_2_ to balance its well‐described pro‐chondrogenic effects against its negative impacts on cell viability 44. As expected, after 24 hours in culture under hypoxic conditions, we detected upregulation of HIF target genes, as others have described 19, 45, as well as increased expression of SOX9 target *COL2A1* and downregulation *COL10A1* (day 14). We also detected an increase in staining for GAGs and reduced Collagen Type X protein formation. Surprisingly, upregulation of *SOX9* was not maintained throughout the 21‐day differentiation. This is in keeping with previous reports that continued upregulation of *SOX9* expression in mouse MSC under hypoxic conditions does not correlate with upregulation of its target genes 11. We also observed that *SOX9* expression was downregulated in the presence of ACF, perhaps suggesting that hBM‐MSC cultures do rely on HIF for physiological hypoxia's downstream effects. Indeed, cells may develop a tolerance to hypoxia following the initial induction 43, and during long‐term culture, hypoxia may act to maintain basal levels of expression of chondrogenic genes.

One of our most striking observations was the ability of DMOG, via HIF‐1α, to induce hBM‐MSC to upregulate expression of HIF target genes and chondrogenic transcripts, and downregulate mRNA encoding hypertrophic chondrocyte markers such as Collagen Type X. In comparison, neither CoCl_2_ nor DFX stimulated similar changes, despite their ability to promote HIF‐1α nuclear localization. The stability and nuclear localization of HIF‐1α is controlled by PHD2, whereas HIF‐1α co‐factor binding is controlled by FIH; DMOG has been shown to inhibit both hydroxylases 22. This is unlike the effect of iron chelators which target PHD2, but do not inhibit FIH as potently 24. Others have shown that FIH requires higher levels of 2‐OG than PHD2 to achieve the same levels of enzymatic activity 46, which may suggest an increased sensitivity of FIH than PHD2 to inhibition by 2‐OG analogs. HIF‐1α activity in hBM‐MSC may also be more dependent on FIH inhibition, rather than PHD2, as high levels of HIF‐1α mRNA have been observed in these cells 42. Indeed, high levels of HIF‐1α transcription might compensate for decreases in HIF‐1α stability due to PHD2‐mediated hydroxylation. Taken together, these observations suggest that regulation of HIF‐mediated transcription that is conducive for hBM‐MSC articular chondrogenesis is dependant more on 2‐OG‐mediated mechanisms than those controlled by intracellular Fe^2+^ levels. Additionally, previous studies which demonstrate the dependence of FIH on 2‐OG availability and the ability of DMOG to inhibit both PHD2 and FIH, suggest that DMOG's potent effect here may be via inhibition of both hydroxylases, whereas CoCl_2_ and DFX may inhibit PHD2 only.

The ability of DMOG to induce an expression profile that is conducive for articular chondrogenesis, suggests its advantage over CoCl_2_ and DFX for use in cartilage‐regenerative therapies. However, despite inducing expression of *COL2A1* and genes involved in post‐translational modifications of collagen, DMOG had a negative effect on cartilage‐like ECM production. We showed that this was partly mediated via HIF‐1α; however, other mechanisms are likely involved as we were unable to completely rescue cartilage‐like ECM formation with Acriflavine. DMOG has been shown to reduce the activity of prolyl‐4‐hydroxylase, which is required for correct folding and polymerization of collagen fibrils 21. Correspondingly, both FIH and collagen prolyl hydroxylase (CP4HA1) have similar affinities for 2‐OG, as they have similar K_m_ values for this co‐factor 47. Therefore, FIH and P4HA1 are likely equally sensitive to DMOG. This suggests that DMOG‐mediated upregulation of HIF target genes via FIH inhibition might be accompanied by a similarly potent inhibition of collagen processing and incorporation into the ECM. Treatment with DMOG for the final 7 days of induction restored the reduced levels of Collagen Type II while upregulating expression of HIF target and chondrogenic genes to similar levels we observed in response to continuous treatment. This response could have been mediated by a lack of continuous inhibition of the collagen prolyl hydroxylase. Taken together, late DMOG treatment, which can stimulate the formation of appropriate ECM, and induce mRNA expression of genes similarly to continuous treatment, may be a valuable strategy for cartilage regenerative medicine.

## Conclusion

Hydroxylase inhibitors are potentially valuable in cartilage tissue engineering strategies as they can mimic many of the effects of hypoxia, providing important environmental cues to progenitors, but without many of its potential drawbacks. Here, we show that CoCl_2_, DFX, and DMOG treatment all induced HIF‐1α stabilization. However, unlike CoCl_2_ and DFX, DMOG treatment strongly regulated HIF targets, and promoted chondrocyte‐specific gene expression. This suggests that in hBM‐MSC undergoing chondrogenic differentiation, HIF‐mediated changes in gene expression may rely more on mechanisms that utilize 2‐OG than those that rely on Fe^2+^. Our observations also suggest a role for DMOG in cartilage tissue engineering strategies. For example, scaffolds that spatially and/or temporally control the release of DMOG could target the articular cartilage to aid in the repair of focal defects. However, the maintenance of cartilage ECM in late treatment‐only conditions suggests the use of this 2‐OG analog would need to be optimized with regard to dosage/treatment time. Alternatively, knowledge that DMOG inhibits both FIH and PHD2 may suggest that dual and specific inhibition of these hydroxylases during de novo cartilage formation may result in HIF‐mediated transcription that is conducive for articular chondrogenesis.

## Author Contributions

D.K.T.: conception and design, collection and assembly of data, data analysis and interpretation, manuscript writing; D.F. and S.F.: collection and assembly of data, manuscript revision; S.L.: provision of hBM‐MSC, manuscript revision; D.I.: conception and design, data analysis and interpretation, manuscript revision; H.W.A.: provision of hBM‐MSC, manuscript revision; A.E.G. and G.J.: conception and design, data analysis and interpretation, manuscript revision; E.G.: conception and design, data analysis and interpretation, manuscript writing, final approval of manuscript.

## Disclosure of Potential Conflicts of Interest

The authors indicated no potential conflicts of interest.

## Supporting information

Supporting Information Table S1

Supporting Information Figure S1

Supporting Information Figures S2

Supporting Information Figures S3

Supporting Information Figures S4
