# Supplementary material for: Differential Regulation of Human Bone Marrow Mesenchymal Stromal Cell Chondrogenesis by Hypoxia Inducible Factor‐1α Hydroxylase Inhibitors
Source: Stem Cells. 2018 Jun 8;36(9):1380–92. doi: 10.1002/stem.2844 (PMC6124654; doi:10.1002/stem.2844)
Supplement: Supplementary file 1 — Supporting Information Table S1 [file STEM-36-1380-s001.docx]

**Supplemental Table 1. qPCR Primer Sequences**

| **Gene of Interest** | **Forward Primer Sequence** | **Reverse Primer Sequence** |
| --- | --- | --- |
| *VEGFA* | AGGGCAGAATCATCACGAAGT | AGGGTCTCGATTGGATGGCA |
| *PGK1* | TGGACGTTAAAGGGAAGCGG | GCTCATAAGGACTACCGACTTGG |
| *EGLN* | AGGCGATAAGATCACCTGGAT | TTCGTCCGGCCATTGATTTTG |
| *SOX9* | AGCGAACGCACATCAAGAC | CTGTAGGCGATCTGTTGGGG |
| *COL2A1* | CCAGATGACCTTCCTACGCC | TTCAGGGCAGTGTACGTGAAC |
| *ACAN* | GTGCCTATCAGGACAAGGTCT | GATGCCTTTCACCACGACTTC |
| *RUNX2* | TGGTTACTGTCATGGCGGGTA | TCTCAGATCGTTGAACCTTGCTA |
| *COL10A1* | GGGGCTAAGGGTGAAAGGG | GGTCCTCCAACTCCAGGATCA |
| *MMP13* | ACTGAGAGGCTCCGAGAAATG | GAACCCCGCATCTTGGCTT |
| *P4HA1* | AGTACAGCGACAAAAGATCCAG | CTCCAACTCACTCCACTCAGTA |
| *LOX* | CGGCGGAGGAAAACTGTCT | TCGGCTGGGTAAGAAATCTGA |
| *RPL13A* | GCCATCGTGGCTAAACAGGTA | GTTGGTGTTCATCCGCTTGC |
