# Supplementary figures and images for: Differential Regulation of Human Bone Marrow Mesenchymal Stromal Cell Chondrogenesis by Hypoxia Inducible Factor‐1α Hydroxylase Inhibitors
Source: Stem Cells. 2018 Jun 8;36(9):1380–92. doi: 10.1002/stem.2844 (PMC6124654; doi:10.1002/stem.2844)

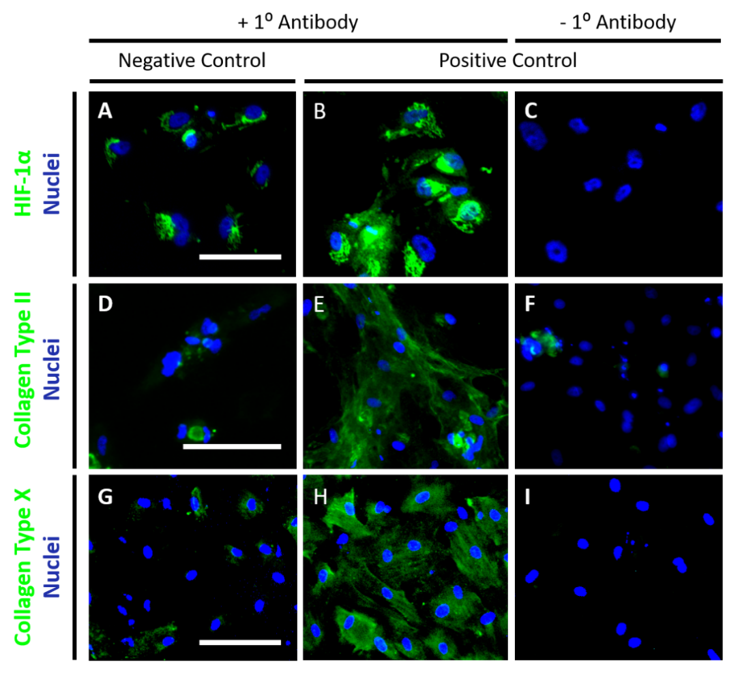

Supplement: Supplementary file 2 — Supporting Information Figure S1 [file STEM-36-1380-s002.tif]

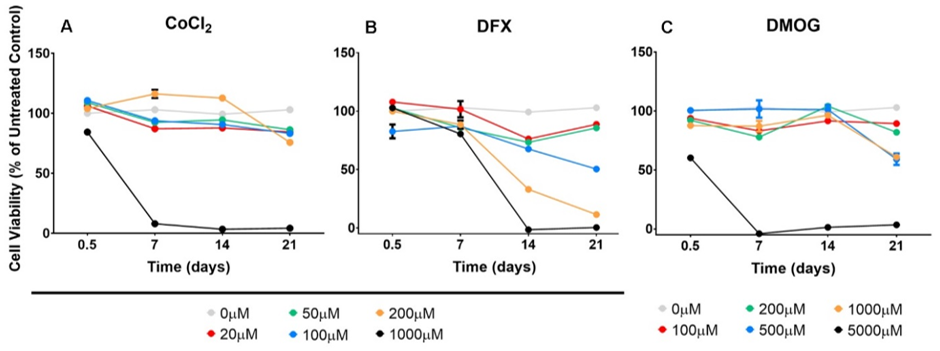

Supplement: Supplementary file 3 — Supporting Information Figures S2 [file STEM-36-1380-s003.tif]

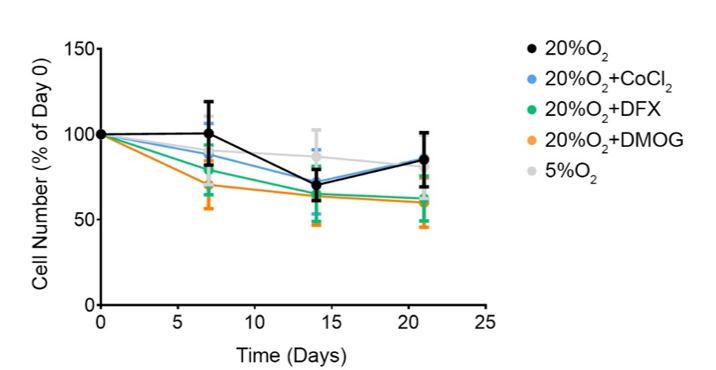

Supplement: Supplementary file 4 — Supporting Information Figures S3 [file STEM-36-1380-s004.tif]

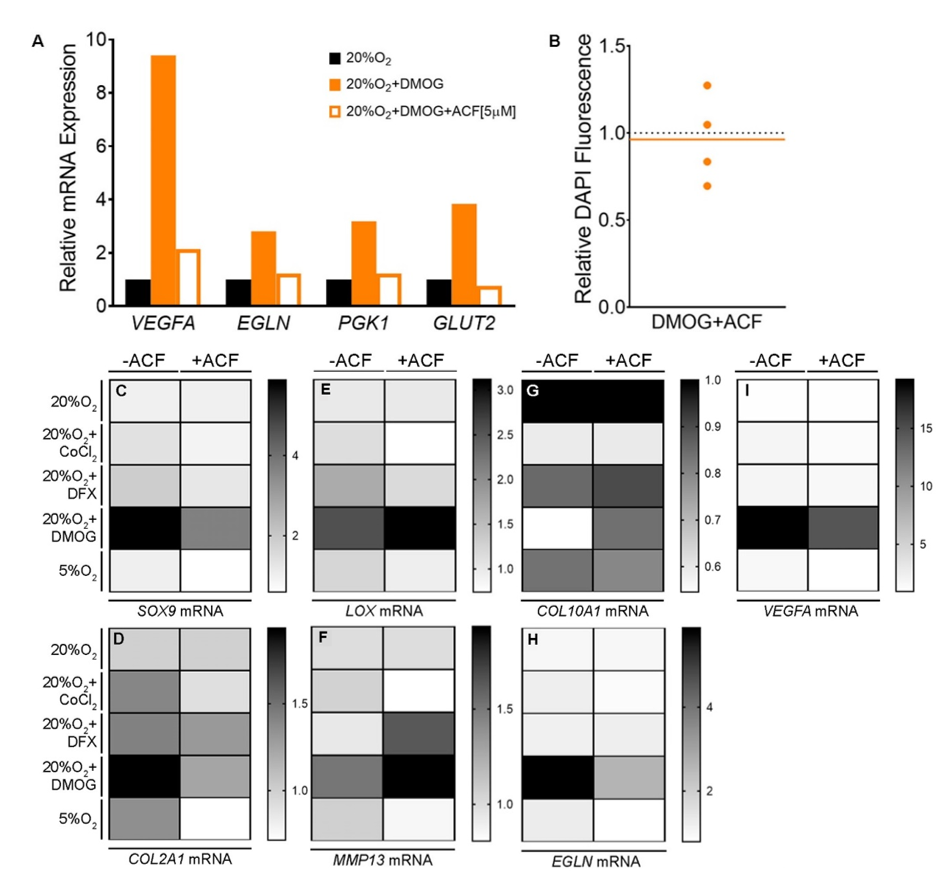

Supplement: Supplementary file 5 — Supporting Information Figures S4 [file STEM-36-1380-s005.tif]
